# Using mass-balance principles to optimize nutrition of lettuce

**DOI:** 10.3389/fpls.2026.1885837

**Published:** 2026-08-10

**Authors:** Noah James Langenfeld, Bruce Bugbee

**Affiliations:** Department of Plants, Soils & Climate, Utah State University, Logan, UT, United States

**Keywords:** deep-water culture, plant nutrition, rootzone, tissue analysis, zero-discharge

## Abstract

The future of agriculture depends on ever more efficient uses of fertilizer inputs. Nutrient management approaches in closed-system hydroponic culture commonly emphasize the need to continuously monitor and control ion concentrations. Here we describe and validate a mass-balance approach that uses the ratio of growth to transpiration (water use efficiency) to calculate nutrient requirements and achieve optimal nutrition without the need for individual ion monitoring. We define optimal nutrition as the minimum fertilizer input to achieve maximum growth. The hydroponic solution volume was maintained by refilling with a nutrient solution in which ion concentrations were calculated based on previously measured leaf nutrient concentrations from healthy plants. Mass-balance recovery of most elements in this closed system was greater than 90%. The yield and nutrient uptake of three diverse lettuce cultivars was consistent over twenty-five consecutive crop cycles (707 days) without replacing any solution. Active absorption of nitrogen (N), phosphorus (P), and potassium (K) led to low and steady solution concentrations of 5 ppm N, 1 ppm P, and 10 ppm K. In the first of three studies, the concentrations of calcium, magnesium, sulfur, boron, and copper increased over time, which indicated that their concentrations were excessive in the refill solution. A high solution concentration did not result in “force feeding” these elements into the plants. Their concentrations were reduced in the second long-term study (seven crop cycles across 196 days) and further reduced in the third long-term study (nine crop cycles across 252 days). These nutrient reductions resulted in constant concentrations and an electrical conductivity of 0.3 mS cm^-1^, which reduced the potential for gaseous N emissions. The third study included a treatment that replaced the nutrient solution after each harvest to maintain an EC of 1.0 mS cm-1. Despite the higher ion concentrations, solution replacement did not increase nutrient uptake or yield, and the increased calcium in solution did not reduce tipburn severity. We conclude that a mass-balance approach can be used to achieve optimal nutrition, maximum yield, and eliminate discharge across multiple crop cycles without continuous monitoring.

## 1 Introduction

Reducing fertilizer and water are essential to optimizing crop production. Under-fertilization reduces yield while over-fertilization can lead to nutrient imbalance, waterbody eutrophication (Daniel et al., 1998), atmospheric emissions of ammonia and nitrous oxide (Govindasamy et al., 2023), and nitrate contamination in ground water (Ward et al., 2018). Excessive fertilization and nutrient leaching are common in field agriculture (Babcock, 1992; Weinbaum et al., 1992; Zhang et al., 2015). Rajsic et al. (2009) found that on-farm fertilization rates were 14% higher than rates recommended by university extension services due to the perception that the low costs of over-fertilization outweighed potential yield loss from under-fertilization.

Containerized plants in soilless media are often over-fertilized and then leached with tap water (Mirmoghadam et al., 2024) or over-fertigated (Xie et al., 2024). Nutrient solutions are frequently discarded and replaced in liquid hydroponics to maintain a set-point electrical conductivity (Miller et al., 2020; Vetrano et al., 2020; Bevan et al., 2021). These practices waste nutrients and create non-steady-state rootzones, which do not reflect the rootzone solution in the field. Excessive fertilizer was recognized as a problem in the 1980s when the Dutch government mandated that all greenhouse facilities work toward zero-discharge practices by the turn of the century (Voogt and Sonneveld, 1997), but the perceived complexity of the approach led to a failure of this mandate.

Excessive fertilization reduces the recovery of nutrients by plants. Nitrogen (N) recovery is of particular significance as volatile nitrous oxide is nearly 300 times more potent than CO_2_ as a greenhouse gas. Govindasamy et al. (2023) reviewed the literature and reported that only about half of the applied N in crop production is recovered in plants. Brown et al. (2025) reported significant volatile losses of N as nitrous oxide and ammonia from containerized production when there were high levels of N in the media. These studies highlight the importance of maintaining low N levels in the rootzone to minimize volatile losses.

### 1.1 Challenges with nutrient management by electrical conductivity

Nutrient solutions can be monitored through electrical conductivity (EC), but ions contribute to EC based on the concentration and square of their charges, so divalent ions contribute four times more to EC than monovalent ions (Griffin and Jurinak, 1973). An initial EC of 1.5 to 3.0 is often maintained by replacing transpired water with a high-EC nutrient solution (Thompson et al., 1998; Son et al., 2020; Liu et al., 2024), but this approach provides no indication of which nutrients remain in solution. Individual nutrients are often over- or under-applied which results in nutrient imbalances. Miller et al. (2020) demonstrated a yield reduction in lettuce when using an EC maintenance approach in a closed system and recommended against its use. They recommended that the solution be discarded after each harvest to reduce the nutrient imbalances.

Nutrients with active uptake are removed from the solution faster than water, which causes the EC to decrease (Bugbee, 2004; Marschner and Marschner, 2012). Maintaining a constant EC therefore leads to the accumulation of nutrients with passive uptake and the depletion of nutrients with active uptake.

Electrical conductivity can be easily and accurately measured in hydroponic solutions and in leachates from soilless media, but it is not possible to interpret the value without an understanding of the input nutrient solution. A low EC does not necessarily indicate a deficiency of nutrients in the root zone. Conversely, a high EC does not indicate adequate nutrients. For example, nitrogen can be deficient, but the EC can be high because of an accumulation of calcium and magnesium. Without an understanding of mass-balance principles, nutrient management by EC can result in non-optimal fertilization.

Another management approach is to continuously monitor the solution concentrations of individual nutrients and adjust nutrient salt or ion inputs to maintain target set points. This instrument-intensive approach, however, requires that controllers and sensors are calibrated and maintained. The concentration for elements with active uptake (N, P, and K) is often less than 10 ppm, which is similar to their concentration in field solutions. Rapid uptake of these elements means that this low set point can be challenging to maintain. The analytical measurement tests (Gavlak et al., 2005) necessary for this approach are expensive and time consuming. Ion-selective electrodes can have interferences from competing ions and need frequent calibrations (Cho et al., 2018; Ahamed et al., 2025). Incorrect interpretation of ion concentration can lead to wasted fertilizer and nutrient imbalances.

### 1.2 Nutrient management by mass balance

A mass-balance-based approach to nutrient management can be used to determine the concentration of nutrients in solution without ion monitoring (Langenfeld et al., 2022). Mass balance is based on the principle that nutrients in a closed system are either in the nutrient solution or plant tissue. The nutrient solution composition is determined by the relative abundance of each nutrient in the plant tissue. The nutrient solution concentration is calculated by multiplying the target tissue nutrient concentration by the rate at which nutrients are removed from solution (water use efficiency, WUE) as shown in Equation 1.

(1) $$\begin{array}{c} tissue\;concentration\;(mɡ\;per\;ɡ)\times WUE\;(ɡ\;per\;L)=solution\;concentration\;(mɡ\;per\;L) \end{array}$$

Solution concentrations can be calculated for all essential nutrients using this approach. Hydroponic systems are fertigated (substrate-based) or refilled (liquid-based) with this nutrient solution each time water is added, and steady-state conditions are maintained. The small, frequent additions of this nutrient solution replace water and deliver only the nutrients necessary for growth.

Nutrient concentrations derived using the mass-balance approach can be refined by measuring the tissue concentration and WUE at the end of each crop cycle. The WUE remains constant if environmental conditions remain constant. Individual nutrient concentrations in the solution can be modified based on measured concentrations in leaf tissue. An increase or decrease of nutrients in the solution that are passively taken up indicates that the either the WUE or tissue concentration estimate need to be adjusted in the mass-balance equation for subsequent nutrient solutions.

### 1.3 Objective

Our objective was to quantify the nutritional and growth effects of repeated plantings of lettuce in a zero-discharge system using a nutrient solution based on mass-balance principles. We hypothesized that this approach would result in consistent yield, healthy tissue nutrient concentrations, and low ion concentrations in the nutrient solution without the need to monitor ions or discard solutions between crop cycles.

## 2 Methods

Three studies were conducted in deep-flow hydroponic culture in a glass greenhouse at the Research Greenhouses at Utah State University in Logan, UT, USA.

### 2.1 Calculating nutrient concentrations in solution

The concentration of elements was based on a WUE of 3 g of dry biomass L^-1^ of water transpired. This is a typical WUE (Langenfeld et al., 2022) for a wide range of plants grown at a CO_2_ concentration of 450 ppm and a relative humidity of 50% (daytime vapor pressure deficit of 1.5 kPa at 25 °C). We multiplied this WUE by the desired tissue concentrations of each nutrient to derive solution concentrations for the initial study (**Table 1**). These solution concentrations are compared to the solution developed by Hoagland and Arnon (1950) in **Table 2**.

**Table 1:** The nutrient salts used to make the initial and refill nutrient solutions in each of the three studies.

Macronutrients	Study 1 (mM)	Study 2 (mM)	Study 3 (mM)	Study 3 (mg L^-1^)
Ca(NO_3_)_2_ • 4 H_2_O	1.5	1.0	0.8	189
KNO_3_	2.0	3.0	3.5→5.0	506
KH_2_PO_4_	0.4	0.4	0.4	54
MgSO_4_ • 7 H_2_O	0.8	0.6	0.5	123
K_2_SiO_3_	0.6	0.6	0.3	46
HNO_3_	1.0	1.0	0.6	38 µL L^-1^
Micronutrients	Study 1 (µM)	Study 2 (µM)	Study 3 (µM)	Study 3 (µg L^-1^)
Fe-DTPA	7	7	7	3909
Mn-EDTA Na_2_ H_2_O	3	3	3	1221
ZnCl_2_	3	3	3	409
H_3_BO_3_	40	16	16	99
Cu-EDTA Na_2_	4	2	2	795
Na_2_MoO_4_ • 2 H_2_O	0.1	0.1	0.1	24
NiCl_2_ • 6 H_2_O	0.1	0.1	0.1	24

Concentrations of some salts were reduced to minimize the gradual accumulation of ions. The calcium nitrate was decreased, and the potassium nitrate was increased in study two and further changed in the second half of study three to ensure adequate nitrogen without excess calcium.

**Table 2:** The elemental concentration in the initial and refill nutrient solutions of each of the three studies compared to the solution developed by Hoagland and Arnon (1950).

Nutrient (mg L^-1^)	Study 1	Study 2	Study 3	Hoagland and Arnon (1950)
N	99	99	**93→116**	210
P	12	12	**12**	31
K	141	180	**176→235**	235
Ca	60	40	**32**	160
Mg	19	15	**12**	49
S	45	38	**16**	64
Si	17	17	**8.4**	–
Fe	0.39	0.39	**0.39**	1.00
Mn	0.16	0.16	**0.16**	0.50
Zn	0.20	0.20	**0.20**	0.05
B	0.43	0.17	**0.17**	0.50
Cu	0.25	0.13	**0.13**	0.02
Mo	0.01	0.01	**0.01**	0.01
Ni	0.002	0.002	**0.002**	–
Cl	0.22	0.22	**0.22**	0.65
Na	0.49	0.40	**0.40**	–

An additional 20% (15 ppm) of nitrogen (N) came from the pH control solution, which also added extra sulfur (17 ppm) in studies 1 and 2. Sodium is not an essential element but comes with the micronutrient chelates and sodium molybdate dihydrate. The N and potassium concentrations were increased in the second half of study three to ensure N availability.

Values in bold font indicate the final, optimal solution concentration that did not result in accumulation of ions.

Each nutrient solution was prepared using reagent grade salts and concentrated (70%) nitric acid, except for chelated iron (Sequestrene 330, BASF Corporation, Research Triangle Park, NC, USA) and potassium silicate (AgSil16H, PQ Corporation, Malvern, PA, USA).

In the second study, the concentrations of calcium (Ca), magnesium (Mg), sulfur (S), boron (B), and copper (Cu) were decreased because they accumulated in the recirculating solution. It was subsequently necessary to increase the concentration of potassium (K) using potassium nitrate to supply adequate nitrogen (N). The concentrations of Ca, Mg, S, and silicon (Si) were further decreased in the third study due to their continued accumulation in the second study.

The N concentration in the nutrient solution in study three decreased by 7 mg L^-1^ because less nitric acid was needed to adjust pH due to the decreased amount of the alkaline potassium silicate. The concentration of potassium nitrate was increased starting with the eighth planting in the third study to ensure adequate N availability.

Silicon was included in the nutrient solution because of its beneficial effects on disease and insect pest resistance (Epstein, 1994).

The whole-plant tissue concentrations were estimated assuming 100% mass balance for each element. This is determined by dividing the elemental concentration in the refill solution by the water use efficiency (**Table 3**). The nutrient solutions were not altered within each study except for an elevation of potassium nitrate in study three as described earlier.

**Table 3:** The whole-plant tissue concentrations that would result from complete mass-balance recovery from solution for each of the three studies.

Nutrient	Units	Study 1	Study 2	Study 3
N	%	3.96	3.96	4.63
P		0.41	0.41	0.41
K		4.69	6.00	6.65
Ca		2.00	1.34	1.07
Mg		0.65	0.49	0.41
S		1.51	1.27	0.53
Si		0.56	0.56	0.28
Fe	mg kg^-1^	130	130	130
Mn		55	55	55
Zn		65	65	65
B		144	58	58
Cu		85	42	42
Mo		3.20	3.20	3.20
Ni		1.96	1.96	1.96
Cl		73	73	73
Na		1.53	1.53	1.53

The tissue concentrations were derived by dividing the refill solution concentrations by a water use efficiency of 3 g L^-1^. These values assume passive uptake, no nutrient exclusion or accumulation in the recirculating nutrient solution and were considered optimal for each element.

### 2.2 Hydroponic system

We used a deep-water culture hydroponic system [described in Langenfeld and Bugbee (2024)] because it its high volume to cultivation area ratio. This provides a larger nutrient buffering capacity compared to other systems, and a more stable concentration of nutrients. This allowed us to document nutrient changes with less experimental error in the measurements.

The system included a 56-L opaque, gray-colored, polypropylene container and a 1-inch-thick sheet of extruded polystyrene covered with white vinyl. If the container volume was reduced, nutrient concentrations would deplete at a faster rate and be more difficult to observe. Eight evenly spaced holes were cut in the polystyrene cover to hold each plant in a neoprene cloning collar. Aeration was continuously provided using outside air at a rate of 5-L of air per minute per container via a 1-inch diameter polyvinyl chloride (PVC) manifold attached to the bottom of each container. The manifold had ten, evenly spaced 2-mm diameter holes to distribute air throughout the nutrient solution.

### 2.3 Plants

Three diverse lettuce cultivars (*Lactuca sativa* cvs. Grand Rapids, Red Sails, and Rex) were germinated on slant boards for seven days using the method described in Langenfeld and Bugbee (2022b). Eight seedlings with uniform root lengths were then transplanted into neoprene cloning collars and placed in the hydroponic system. Each cultivar grew as a community of eight plants with one cultivar per container (n = 1) as described in Langenfeld and Bugbee (2024).

### 2.4 Environmental conditions

The temperature was controlled at a 25/20 °C day/night split. The relative humidity was not controlled but averaged 50% ± 20% throughout the studies. The daily light integral (DLI) varied due to seasonal changes, but supplemental LED lighting on a 16-hour photoperiod ensured the photosynthetic photon flux density (PPFD) remained above 400 µmol m^-2^ s^-1^ during the day to provide a DLI that ranged from 25 to 40 mol m^-2^ d^-1^ throughout the year. This DLI is higher than what is typically used in commercial production but was selected to induce rapid growth and transpiration to create a more challenging environment for nutrient management.

The first study was over twenty-five consecutive, 28-day crop cycles across 707 days (January 2022 to March 2024), the second study was over seven consecutive, 28-day crop cycles across 196 days (January to August 2023), and the third study was over nine consecutive, 28-day crop cycles across 252 days (March to December 2024).

### 2.5 Nutrient solution management

The electrical conductivity (EC) was measured every day before refilling using an EC meter (model DiST 3, Hanna Instruments, Woonsocket, RI, USA). The meter was calibrated at the beginning of each study using a 1410 µS cm^-1^ standard and maintained calibration throughout the study durations. The nutrient solution was refilled as needed to maintain at least 90% of the initial solution volume (53 L). The refill solution had the same nutrient composition and concentration as the initial solution within each study. *No nutrient solution was discarded in any of the three long-term studies – water and nutrients were only removed by plants via root uptake and the transpiration stream.* The closed cover reduced evaporation of solution to less than 1% of transpiration (Langenfeld et al., 2022). The nutrient solution was retained following each harvest, and new, 7-day-old seedlings were transplanted into the same solution. This process was repeated for twenty-five plantings in study one, seven plantings in study two, and nine plantings in study three. The third study included a solution replacement (SR) treatment for each cultivar where the nutrient solution was completely replaced with fresh solution following each harvest.

### 2.6 Automated pH control

The pH of each rootzone was controlled using an automated pH control system (Langenfeld and Bugbee, 2022a, 2024). A pH electrode (model UX-59001-65, Cole-Parmer, Vernon Hills, IL, USA) was attached to a 12-volt pH controller (model BL931700-0, Hanna Instruments, Woonsocket, RI, USA) set to maintain the pH at 5.8 through automated additions of a solution of 50 mM nitric acid and 25 mM ammonium sulfate (100 mM total N) via a solenoid pinch valve (model SwitchEZ, Precigenome LLC, San Jose, CA, USA) connected to a gravity-fed reservoir. A time delay relay was installed to open a valve for 5 s, which transferred about 20 mL of pH control solution into the hydroponic container at each dosing event. In the third study the pH control solution was changed from ammonium sulfate to ammonium nitrate to reduce sulfate accumulation in the nutrient solution.

### 2.7 Harvest procedure

After 28 days (35 days after seeding), plants were harvested and the fresh mass of roots and shoots was measured. Tipburn was quantified in study three using the tipburn index developed by Frantz et al. (2004). The plant tissue was then separated into three categories: young leaf tissue (youngest, innermost leaves near the meristem, 3 to 5 g per plant), mature leaf tissue (all other leaves not considered young, study three only), and root tissue. Dry mass was measured after drying the tissues in an 80 °C oven to constant mass (2 to 5 d). Each complete dry tissue portion was ground in a mortar and pestle, and a 10 g sample was taken for nutrient analysis. This approach allowed average nutrient concentrations to be calculated for each part of the plant and a complete mass-balance recovery to be calculated in study three.

### 2.8 Tissue and solution analysis

Dry tissue samples were separately digested in 15.7 M nitric acid and 30% hydrogen peroxide [method B-4.25 in Gavlak et al. (2005)] and analyzed using inductively coupled plasma optical emission spectroscopy (ICP-OES) by the Utah State University Analytical Laboratory in Logan, UT, USA. Nitrate-N was measured by digesting tissue samples in 27 mM calcium hydroxide and analyzing them with the cadmium reduction method for flow injection analysis (QuikChem Method 12-107-04-1-C). Dry tissue samples were analyzed for total N using combustion [method B-2.20 in Gavlak et al. (2005)]. The elemental concentrations in the nutrient solution were determined using ICP-OES without acid pretreatment. Nitrate-N in the nutrient solution was also analyzed using cadmium reduction [method W-1.80 in Gavlak et al. (2005)].

### 2.9 Mass-balance recovery of each element

Mass-balance recovery of each element was calculated for study three. The input nutrient mass was calculated by multiplying the nutrient concentration in the fresh nutrient solution by the total volume of solution added to the container over the planting. This value was added to the nutrients contained in the container before planting [the concentration remaining after the previous harvest multiplied by 53 L (the container solution volume)]. The solution concentration from the previous harvest was the same as the fresh solution concentration in the first planting. The amount of N contributed from the pH control solution was included in the input of N.

The remaining nutrients in solution were calculated by multiplying the nutrient concentration in the remaining solution after harvest by the volume of remaining nutrient solution. The amount contained in the plant tissue was calculated by multiplying the nutrient concentration in each plant tissue sample by the dry mass of the tissue from which the sample was taken. The nutrients contained in the solution and tissues after harvest were summed, divided by the input nutrients, and multiplied by 100% to calculate a mass-balance recovery (in percent) for each nutrient as shown in Equation 2. The mass-balance recovery was calculated for all essential elements in the nutrient solution except nickel (trace amount) and chlorine (no analysis conducted).

(2) $$\frac{\left[remaining\;solution\;(\frac{mɡ}{L})\times remaining\;volume\;(L)]+[young\;leaf\;(\frac{mɡ}{kɡ})\times young\;leaf\;(kɡ)]+[mature\;leaf\;(\frac{mɡ}{kɡ})\times mature\;leaf\;(kɡ)]+[root\;(\frac{mɡ}{kɡ})\times root\;(kɡ)\right]}{\left[fresh\;solution\;(\frac{mɡ}{L})\times total\;volume\;added\;(L)]+[solution\;after\;previous\;harvest\;(\frac{mɡ}{L})\times 53\;L\right]}\times 100\%$$

## 3 Results

**Figure 1:**
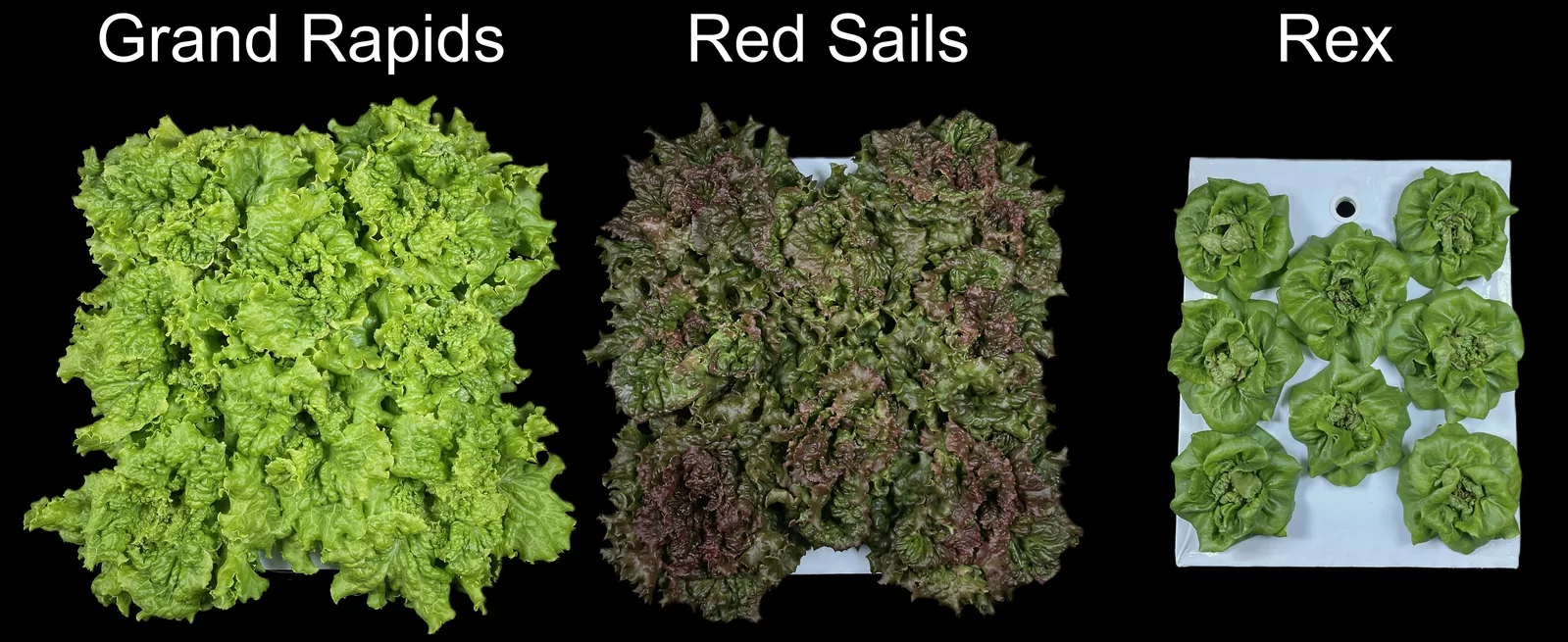
Top view of the three lettuce cultivars (Grand Rapids, Red Sails, and Rex) prior to the first harvest in study three. This photograph is typical of the plants at each harvest throughout all studies. Some tipburn is apparent in the young leaves of the cultivar Rex.

The three cultivars were diverse (**Figure 1**). Grand Rapids and Red Sails are loose-leaf cultivars. Red Sails has purple leaves from its elevated anthocyanin content (Zhang and Folta, 2012). Rex is a butterhead cultivar and has a slower growth rate than Grand Rapids and Red Sails. All three cultivars grew well in all studies. We observed bright white roots and optically clear nutrient solutions with a turbidity of zero Nephelometric Turbidity Units (NTU, data not shown) throughout repeated plantings.

### 3.1 Calculation of water use efficiency

**Figure 2:**
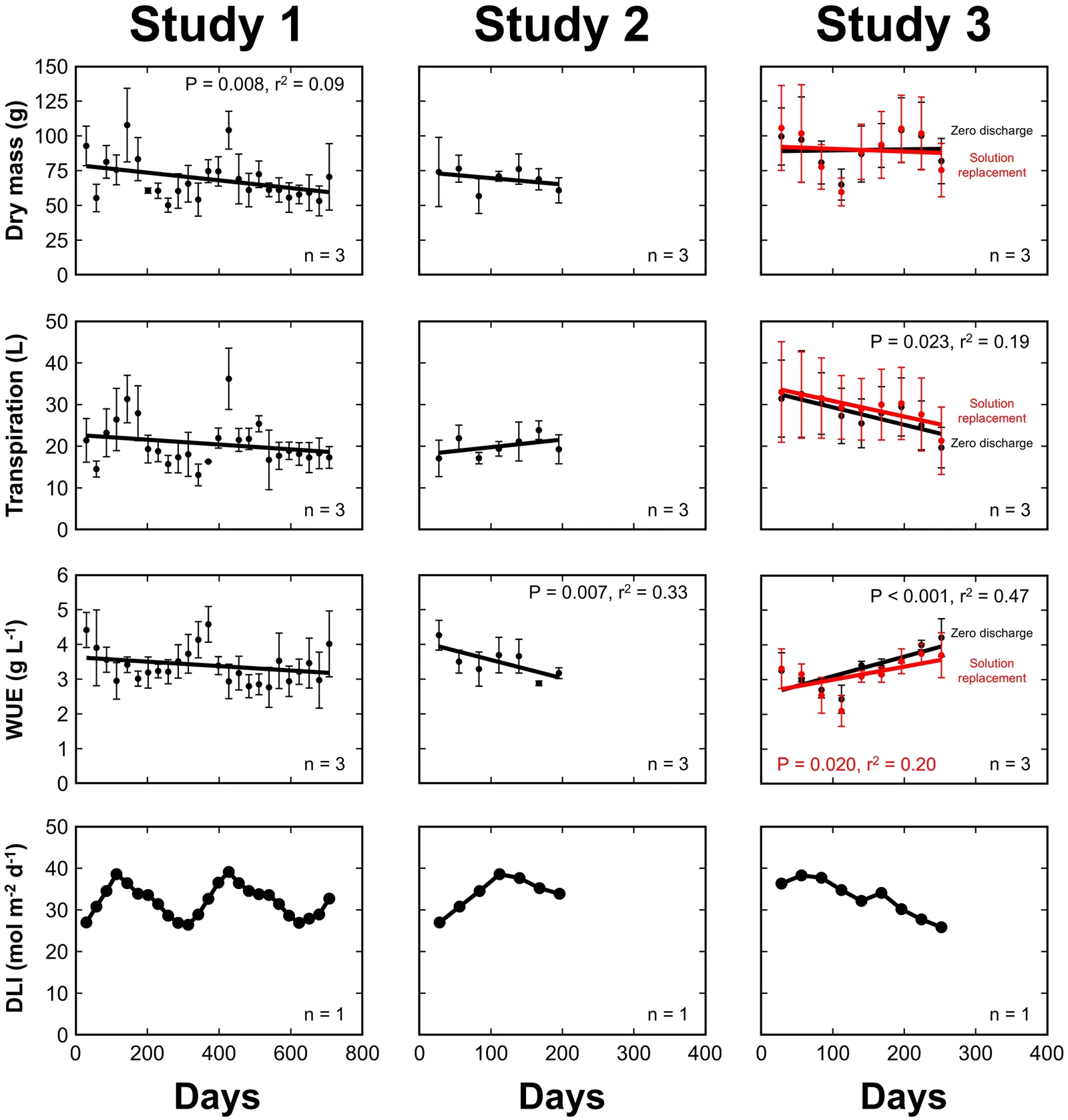
The dry mass, transpiration, water use efficiency, and daily light integral across repeated plantings in three separate studies. The red lines in study three indicate containers where the nutrient solution was completely replaced following each harvest. Error bars represent standard deviation, n = 3 lettuce cultivars. Each trend was analyzed for statistical significance among pseudoreplicates, but statistics are only included for significant differences to improve figure clarity.

The total dry mass (shoots and roots) from each harvest and cultivar was divided by the cumulative transpiration to determine the WUE (**Figure 2**). The dry mass, transpiration, and WUE were the most variable in study one and the least variable in study three. Grand Rapids typically had the highest dry mass, and Rex typically had the lowest dry mass. Red Sails tended to have the lowest WUE while Rex tended to have the highest WUE. Transpiration changes were correlated with seasonal changes in daily light integral (DLI). There was no difference in study three among any of the measured parameters between plants grown using a zero-discharge or dump and refill management approach.

The photon conversion efficacy [PCE (Westmoreland et al., 2021)] was calculated by dividing yield (grams m^-2^ d^-1^) by the DLI (moles of photons m^-2^ d^-1^). In study one, the PCE averaged 0.2 g of dry biomass per mol of photons, and there was no change in PCE over time (**Supplementary Figure 1**). There was a slight decrease in PCE across study two and a slight increase in PCE across study three. Neither the PCE within a crop cycle, nor the trend in PCE over time, were significantly different between the zero-discharge the solution refill approaches.

### 3.2 Cultivar and management effects on tipburn

**Figure 3:**
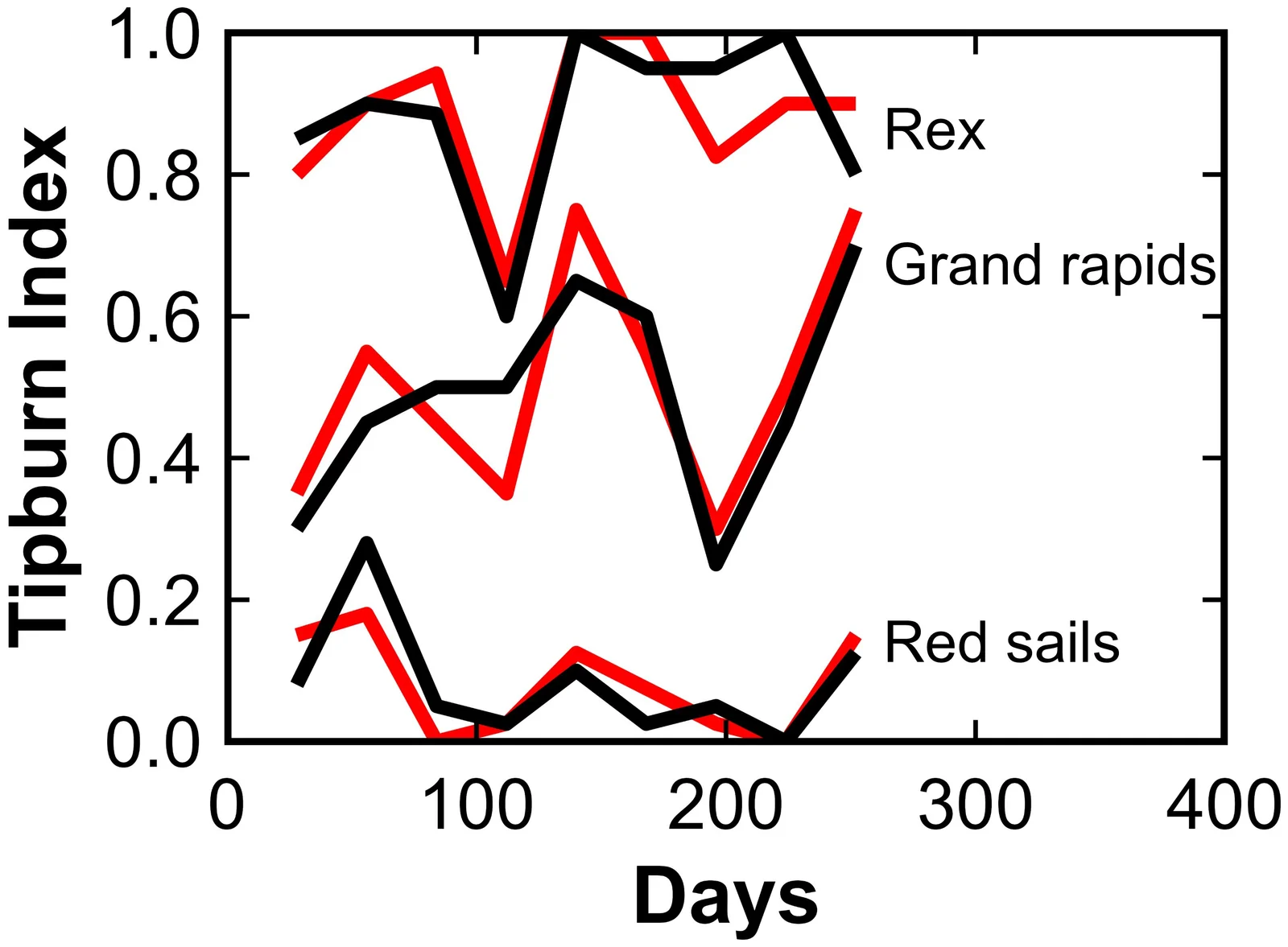
The tipburn severity index in study three over the nine repeated plantings in three lettuce cultivars: Grand Rapids, Red Sails, and Rex. The black lines indicate the treatments with solution recycling (zero-discharge), and the red lines indicate that the nutrient solutions were completely replaced following each harvest (solution replacement). The calcium concentration in the nutrient solutions was about three times higher in the solution replacement treatment, but this did not reduce tipburn in any cultivar.

Tipburn was evaluated at each harvest in study three (**Figure 3**). There was no significant difference between management approaches on tipburn, but there were significant cultivar differences: Rex consistently had the highest tipburn index (0.8) while Red Sails consistently had the lowest tipburn index (0.1).

### 3.3 Electrical conductivity

**Figure 4:**
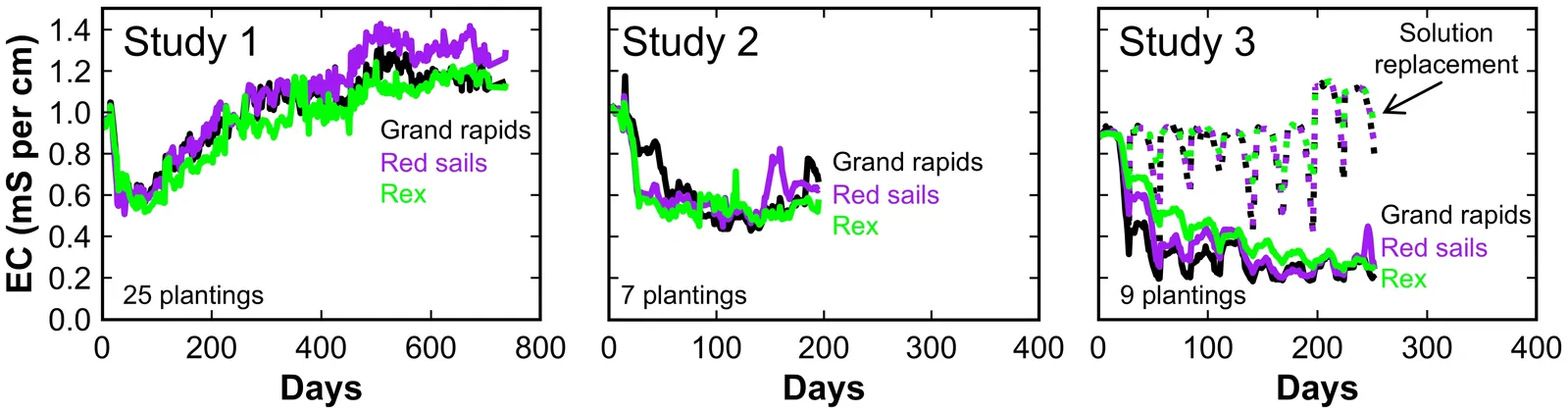
The electrical conductivity (EC) of the hydroponic nutrient solution over the course of twenty-five plantings (study one), seven plantings (study two), and nine plantings (study 3) of three lettuce cultivars: Grand Rapids (black), Red Sails (purple), and Rex (green). The solid lines indicate that nutrient solutions were reused after each harvest (zero-discharge), and the dashed lines indicate the nutrient solutions were completely replaced following each harvest (solution replacement). As expected, the EC declined during the first planting as nutrients with active uptake were removed.

The initial nutrient solution EC was 1.0 mS cm^-1^ in study one and study two. The initial EC in study three was 0.9 mS cm^-1^ due to the reduced Ca and S concentrations. The EC decreased to between 0.4 to 0.6 mS cm^-1^ by the end of the first harvest in each study (**Figure 4**). In study one, the EC slowly increased to about 1.2 mS cm^-1^ after about 500 days (18 plantings) and remained steady for future plantings. The EC remained at about 0.6 mS cm^-1^ in study two and ranged from 0.2 to 0.4 mS cm^-1^ in study three across plantings. The EC was reset to 0.9 mS cm^-1^ with fresh solution at the start of study three. The cultivar Rex had the slowest increase in EC in study one and slowest decrease in EC in study three, which was correlated with its slower growth rate compared to Grand Rapids and Red Sails. The EC spikes observed in study two were caused by the failure of two pH electrodes that led to extra acid being added to each tank. This was corrected by adding potassium hydroxide, which temporarily increased the EC.

### 3.4 Nutrient solution concentrations

**Figure 5:**
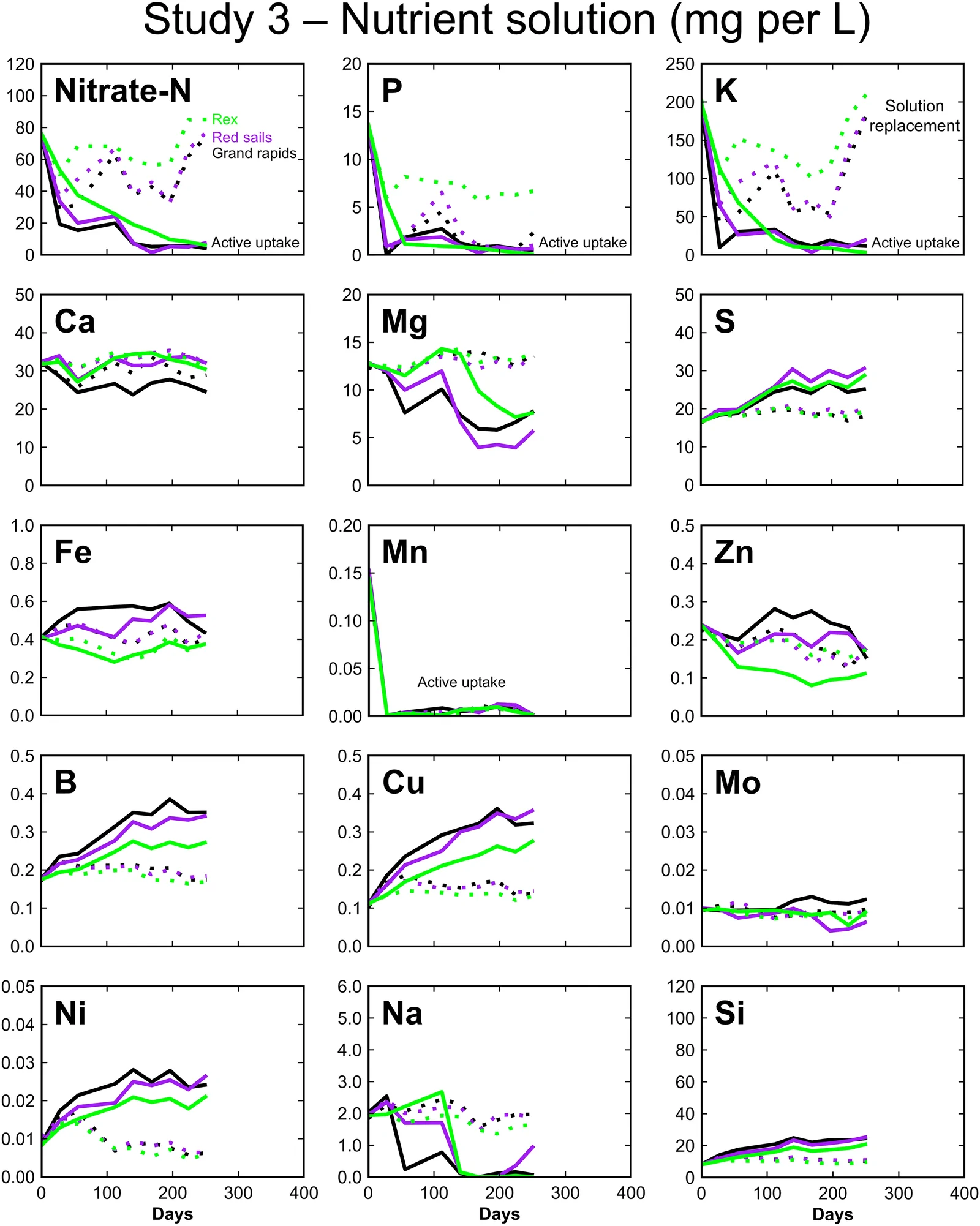
The change in the solution concentration of nutrients in study three over the course of nine repeated plantings in three lettuce (*Lactuca sativa*) cultivars: Grand Rapids (black), Red Sails (purple), and Rex (green). The dashed lines indicate containers where the nutrient solution was completely replaced after each harvest, and the solid lines indicate treatments where the bulk nutrient solution was retained after each harvest.

The nutrients remaining in solution at the end of each planting aligned with the categories of established nutrient uptake rates. Nutrients with active uptake (N, P, and K) were rapidly removed from the nutrient solutions during each planting in studies one and two (**Supplementary Figures 2**, **3**). Manganese also decreased rapidly in the nutrient solution after the first harvest in each study. All other nutrients with intermediate or passive uptake were stable in solution over time or increased. The rate of increase of Ca, Mg, S, B, Cu, and Si concentration was reduced in study two and further reduced in study three (**Figure 5**). There was no consistent pattern of sodium accumulation or removal in any study.

### 3.5 Tissue concentration in young leaves

**Figure 6:**
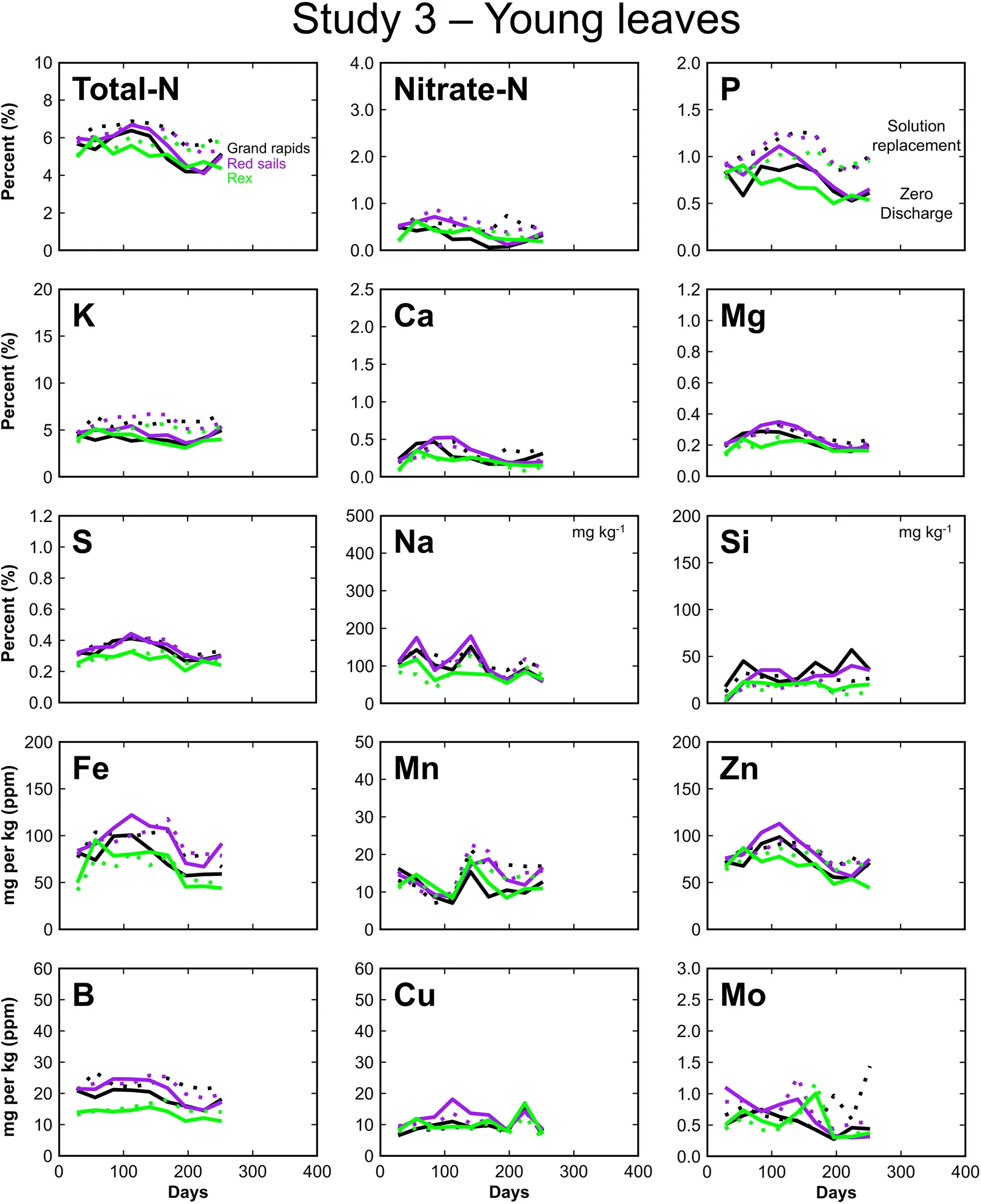
The change in the young leaf tissue concentration of essential nutrients in study three over the course of nine repeated plantings in three lettuce (*Lactuca sativa*) cultivars: Red Sails (purple), Rex (green), and Grand Rapids (black). The dashed lines indicate containers where the nutrient solution was completely replaced after each harvest, and the solid lines indicate treatments where the bulk nutrient solution was retained after each harvest.

There was no increasing or decreasing trend over time in the concentration of any element in young leaf tissues in studies one and two (**Supplementary Figures 4**, **5**), but there were small differences between the solution replacement and zero-discharge treatments in study three (**Figure 6**). Containers in the solution replacement treatment tended to have excessive levels of N, P, and K in the young leaves. There were slight differences among cultivars. Red Sails tended to have the highest nutrient concentrations while Rex tended to have the lowest nutrient concentrations.

### 3.6 Tissue concentration in mature leaves

**Figure 7:**
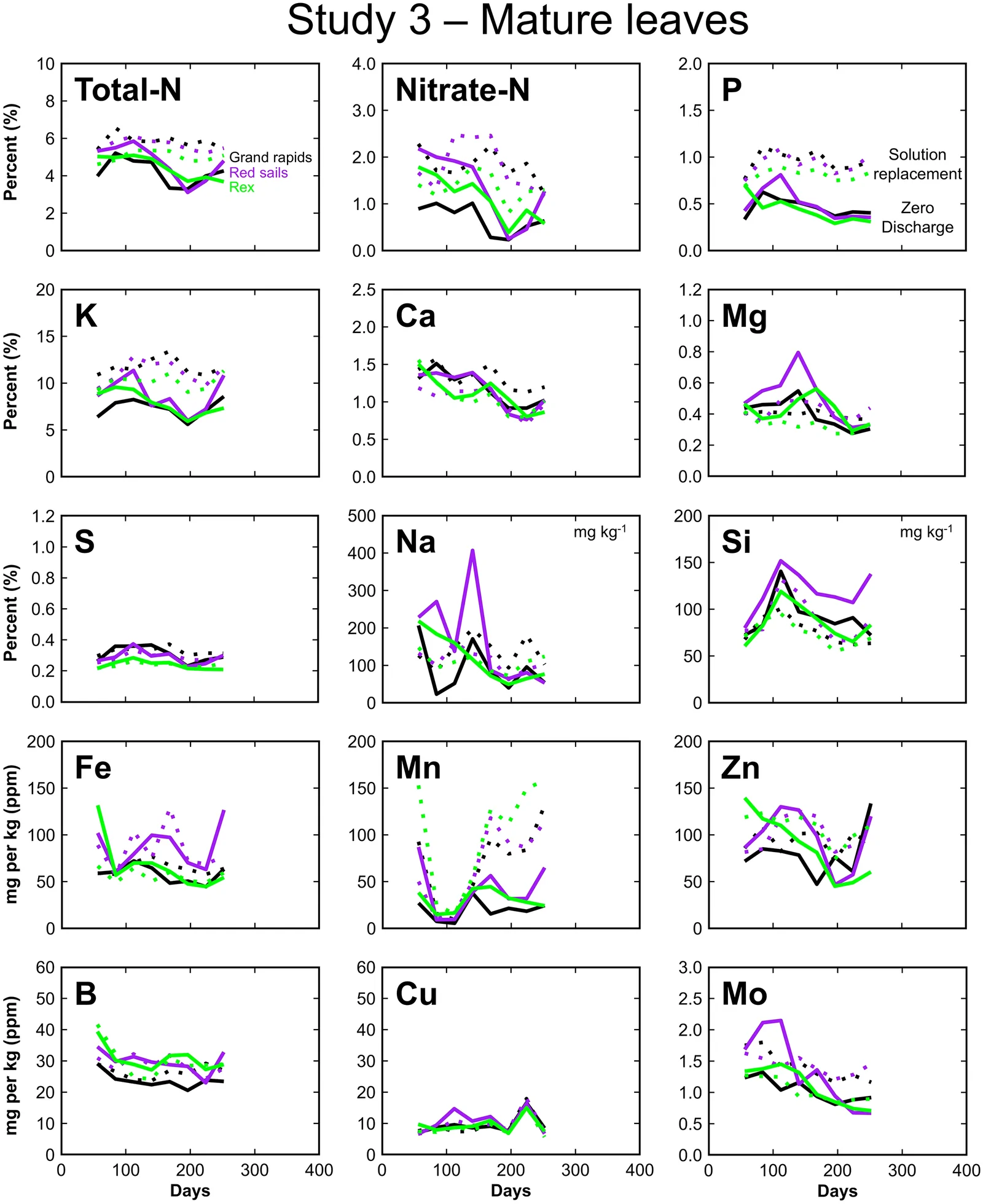
The change in the mature leaf tissue concentration of essential nutrients in study three over the course of nine repeated plantings in three lettuce (*Lactuca sativa*) cultivars: Red Sails (purple), Rex (green), and Grand Rapids (black). Samples were not analyzed after the first harvest. The dashed lines indicate containers where the nutrient solution was completely replaced after each harvest, and the solid lines indicate treatments where the bulk nutrient solution was retained after each harvest.

The nutrient concentrations in the mature leaf tissues were measured starting with the second planting in study three (**Figure 7**). The trends in the mature leaf tissue generally matched those found in the young leaf tissue. The sharp increases and decreases in copper and sodium observed in the mature leaf tissue were also observed in the young leaf tissue. The concentrations of nitrate-N, K, Ca, Mg, and Si were higher in the mature leaf tissue than the young leaf tissue. The Fe, Mn, and Zn concentrations were erratic among harvests.

### 3.7 Tissue concentration in roots

**Figure 8:**
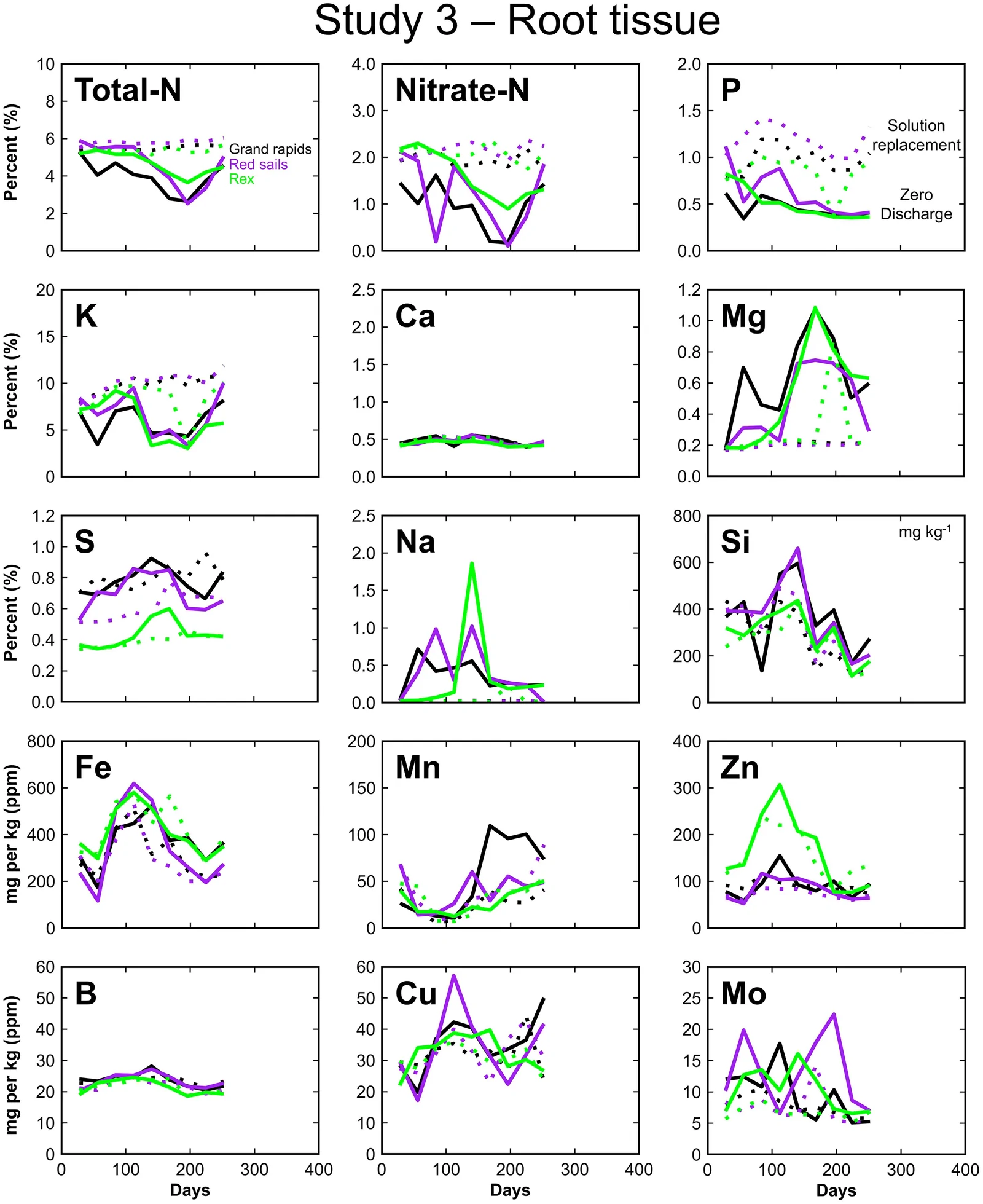
Change in the root tissue concentration of essential nutrients in study three over the course of nine repeated plantings in three lettuce (*Lactuca sativa*) cultivars: Red Sails (purple), Rex (green), and Grand Rapids (black). The dashed lines indicate containers where the nutrient solution was completely replaced after each harvest, and the solid lines indicate treatments where the bulk nutrient solution was retained after each harvest.

The nutrient concentrations in the root tissue in each study were generally lower than in the leaf tissue for nutrients with active uptake (N, P, and K) and higher for nutrients with passive uptake. The concentrations among repeated plantings were also more variable in the root than the leaf tissue. The B concentration tended to increase across plantings in study one (**Supplementary Figure 6**), but there were no trends among other nutrients or studies. We measured stable concentrations of B and Ca among harvests in studies two (**Supplementary Figure 7**) and three (**Figure 8**), but the other nutrient concentrations were more variable among repeated harvests. Plants grown under the solution replacement approach in study three tended to have higher concentrations of N, P, and K than those under the zero-discharge management scheme. The sodium concentration was an order of magnitude higher in the root tissue than leaf tissues in all studies, which indicated exclusion of sodium on root surfaces.

### 3.8 Whole plant tissue concentrations

Whole-plant tissue concentrations (weighed average of young leaf, mature leaf, and root concentrations) were calculated for plantings two through nine in study three (**Table 4**). There was no significant difference in tissue concentrations among plantings, so concentrations were pooled for statistical analysis (n = 8). Potassium was the element with the highest concentration in the plant tissue among all three cultivars and averaged 10.4% in plants grown with the solution replacement approach and 7.7% in plants grown in zero-discharge solutions. Rex tended to have lower tissue concentrations for macronutrients compared to Grand Rapids and Red Sails. We used an ANOVA with Tukey’s HSD test to compare treatments within cultivar and found significant differences (p < 0.05) with N, P, and K in Grand Rapids; P, K, Si, and Mo in Red Sails; and P, K, and Mn in Rex.

**Table 4:** The whole plant tissue concentrations of three lettuce cultivars (Grand Rapids, Red Sails, and Rex) grown in deep flow hydroponics where the nutrient solution was either replaced (solution replacement, SR) or retained (zero-discharge, ZD) after every harvest.

Element	Unit	Grand Rapids		Red Sails		Rex		Average	
		SR	ZD	SR	ZD	SR	ZD	SR	ZD
N	%	5.6*	4.2*	5.5	4.7	4.8	4.2	5.3	4.4
P		1.0*	0.5*	1.0*	0.6*	0.8*	0.5*	0.9	0.5
K		11.1*	7.3*	10.7*	8.4*	9.3*	7.3*	10.4	7.7
Ca		1.2	1.0	0.9	1.0	0.9	0.9	1.0	1.0
Mg		0.4	0.4	0.4	0.5	0.3	0.4	0.4	0.4
S		0.4	0.4	0.3	0.3	0.3	0.3	0.3	0.3
Si	ppm	83	105	94*	123*	76	87	84	105
Fe		97	106	106	120	104	114	102	113
Mn		57	27	61	34	77*	31*	65	31
Zn		92	82	95	94	111	97	99	91
B		26	24	28	28	27	27	27	26
Cu		12	13	12	14	11	13	12	13
Mo		2.2	2.3	2.0*	2.9*	1.7	2.3	2.0	2.5

The only significant differences between management schemes within cultivar were N, P, and K in Grand Rapids; P, K, Si, and Mo in Red Sails; and P, K, and Mn in Rex. These significant differences (n = 8) are identified with an asterisk. The higher concentrations are considered to be luxury uptake as there was no growth benefit.

### 3.9 Nutrient distribution among plant tissues

The nutrient distribution among young leaf, mature leaf, and root tissue varied among elements (**Table 5**). There were no significant differences in tissue distribution among cultivars or plantings in study three, so measurements were pooled for analysis. The tissue concentrations in the first planting were excluded from this analysis because mature leaf tissue was not analyzed. Plants grown in solution replacement treatments often had lower concentrations of macronutrients than plants grown in zero-discharge treatments. We observed the opposite trend for many micronutrients. The concentrations of Ca and S in young and mature leaf tissue, and in the root tissue, were similar between the two treatments.

**Table 5:** The distribution of elements in the plant tissues for eight harvests from study three.

Element	Unit	Young leaves		Mature leaves		Roots	
		SR	ZD	SR	ZD	SR	ZD
N	%	5.4	4.7	4.9	4.0	5.0	3.8
P		0.9	0.7	0.8	0.4	0.9	0.4
K		5.0	3.8	9.8	7.1	8.6	5.4
Ca		0.3	0.3	1.0	1.0	0.4	0.4
Mg		0.2	0.2	0.3	0.4	0.2	0.5
S		0.3	0.3	0.3	0.3	0.5	0.6
Si	ppm	21	29	71	88	129	162
Fe		75	71	61	63	310	339
Mn		13	11	71	26	27	36
Zn		70	66	89	78	96	102
B		18	16	25	25	20	21
Cu		9	10	8	9	28	31
Mo		0.6	0.5	1	1	6	10

Cultivars were pooled for analysis. Each value represents an average of 24 pooled measurements (three cultivars and eight harvests). The nutrient solution was either replaced (solution replacement, SR) or retained (zero-discharge, ZD) after every harvest.

Nutrient ratios among tissues are shown in **Table 6**. The highest accumulations of nutrients in the mature leaf tissue relative to the young leaf tissue in the solution replacement treatment occurred with Mn (5.65-fold), Ca (4.13-fold), and Si (3.41-fold). The highest accumulations of nutrients in the root tissue relative to the young leaf tissue in the solution replacement treatment occurred with Mo (10.93-fold), Si (6.19-fold), and Fe (4.15-fold).

**Table 6:** The effect of solution replacement on the ratio of nutrients among plant tissues.

Element	Solution replacement			Zero-Discharge		
	Young leaves	Mature leaves	Roots	Young leaves	Mature leaves	Roots
N	1	0.91	0.93	1	0.84	0.81
P	1	0.88	0.99	1	0.62	0.65
K	1	1.93	1.69	1	1.87	1.42
Ca	1	4.13	1.74	1	4.11	1.67
Mg	1	1.63	1	1	1.93	2.57
S	1	0.84	1.82	1	0.86	2.03
Si	1	3.41	6.19	1	3.04	5.63
Fe	1	0.81	4.15	1	0.88	4.76
Mn	1	5.65	2.16	1	2.38	3.34
Zn	1	1.27	1.36	1	1.18	1.54
B	1	1.40	1.11	1	1.61	1.32
Cu	1	0.96	3.33	1	0.90	3.20
Mo	1	1.94	10.93	1	2.04	19.78

Data are from eight harvests in study three. Cultivars were pooled for analysis. The ratios were calculated from the data in **Table 5**. The nutrient solution was either replaced (solution replacement, SR) or retained (zero-discharge, ZD) after every harvest.

The highest accumulations of nutrients in mature relative to the young leaf tissue in the zero-discharge treatment occurred with Ca (4.11-fold), Si (3.04-fold), and Mn (2.38-fold). The highest accumulations of nutrients in root tissue relative to the young leaf tissue in the zero-discharge treatment occurred with Mo (19.78-fold), Si (5.63-fold), and Fe (4.76-fold).

### 3.10 Mass-balance recovery

The mass-balance recovery of each element was determined in study three in both the solution replacement and zero-discharge treatments. Mature leaf tissue was not analyzed in studies one and two, so mass-balance recovery was not calculated. The mature leaf tissue was not analyzed in the first harvest of study three due to a harvesting error, and the solution was not analyzed after the third harvest of study three. We therefore estimated the mature leaf tissue concentration from the first harvest by using an average mature leaf tissue concentration measured in subsequent plantings. The low variability in tissue concentrations across plantings confirmed the applicability of this approach. Nutrient solution concentrations over time followed similar patterns within studies, and averaging the data from any two harvests separated by a single harvest closely agreed with the actual data. We therefore estimated the solution concentration after the third harvest in the third study by interpolating between the second and fourth plantings within that study.

**Figure 9:**
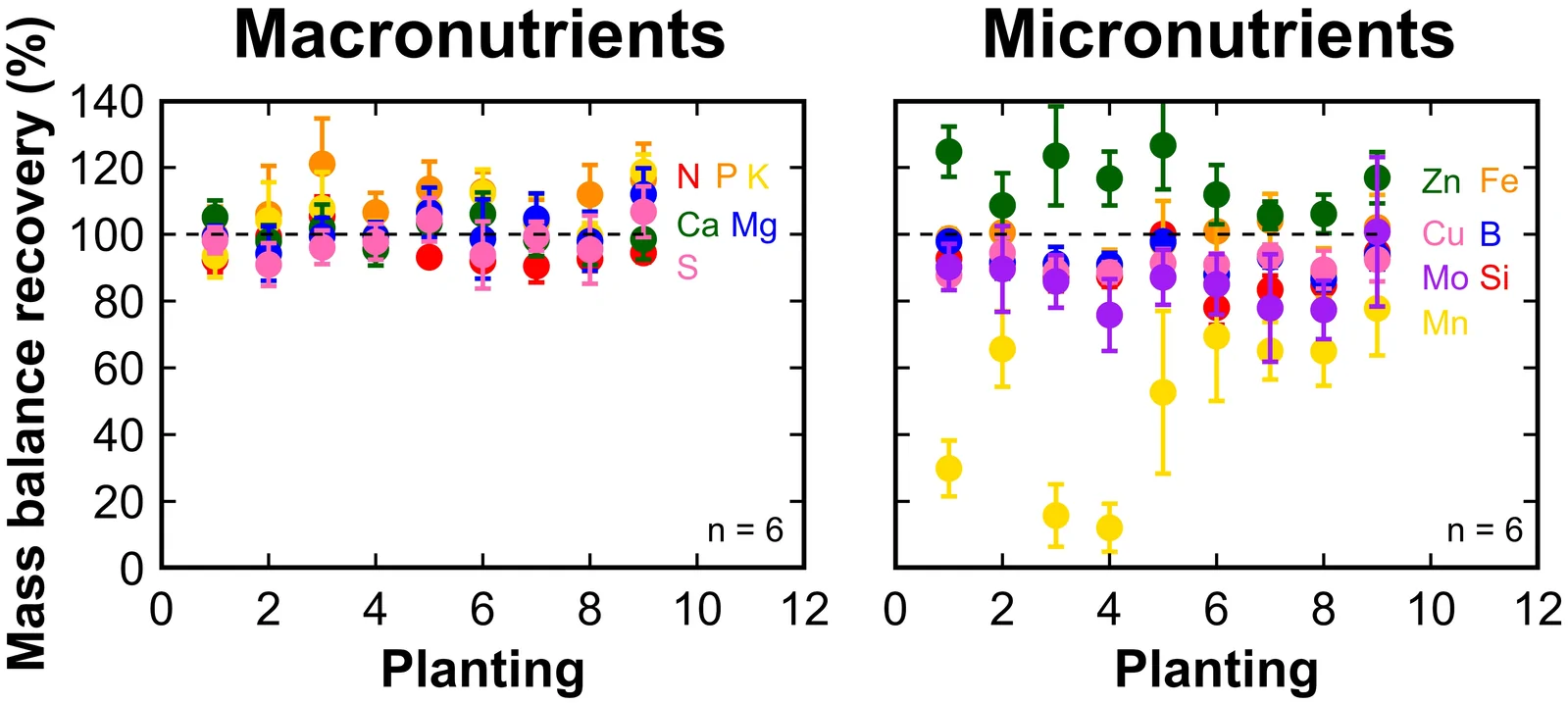
The mass-balance recovery of nutrients for three lettuce cultivars grown in nine repeated plantings. Recovery was calculated by dividing the sum of each nutrient in the plant tissue and nutrient solution at harvest by the amount of each nutrient applied in the nutrient solution over the course of each growth cycle. Recoveries among cultivars and treatments were combined. Error bars represent standard deviation, n = 6. All recoveries were 100% ± 10%, except zinc (116%), Si (88%), Mo (86%), and Mn (50%).

There was no difference in mass-balance recovery between treatments, so they were pooled for analysis. Most elements had a mass-balance recovery of 100 ± 10% (**Figure 9**). Zinc had an average recovery of 116%, silicon had a recovery of 88%, molybdenum had a recovery of 86%, and manganese a recovery of 50%.

## 4 Discussion

The results presented here provide no indication of a difference in yield, WUE, or mass-balance recovery between solution replacement and zero-discharge nutrient management approaches.

### 4.1 Yield across replicate crop cycles

The lack of differences in tissue concentrations over time indicates that nutrition was not limiting. There was also no difference in yield or tissue concentrations with solution replacement in study three, indicating that an EC of 0.3 mS cm^-1^ was adequate for maximum growth.

Yield differences among plantings were correlated with daily light integral. Supplemental lighting maintained a DLI above 25 mol m^-2^ d^-1^, which is higher than commercial levels and higher than most lettuce research (Gavhane et al., 2023). This high DLI resulted in rapid growth but likely increased tipburn severity. Frantz et al. (2004) found that yield increased up to a DLI of 57 mol m^-2^ d^-1^ when tipburn was minimized. Kelly et al. (2020) saw a linear increase in lettuce growth as DLI increased from 6.9 to 15.6 mol m^-2^ d^-1^ and Gavhane et al. (2023) reported an increase in the photosynthetic rate of lettuce up to a PPFD of 1000 µmol m^-2^ s^-1^. These studies show that higher light intensities can drive higher yields. In our work, the photon conversion efficacy was consistent among studies, indicating that lettuce growth responds to a high DLI.

### 4.2 Water use efficiency

Variation in WUE among plantings within studies may have been caused by incomplete drying of plant biomass. Packing density within drying bags may have restricted airflow and could have led to constant masses that were not uniformly dry. Despite this potential drying variability, there were only minor measured and perceived differences in WUE across crop cycles.

There was a cultivar difference in WUE. Our results are similar to those obtained by Clavijo-Herrera et al. (2018), who reported a significantly lower WUE in the red-leafed cultivar ‘Cherokee’ compared to the green-leafed cultivar ‘Waldmann’s Green’. Red Sails tended to have a lower WUE than Grand Rapids, which could be due to its higher anthocyanin concentrations and reduced photosynthetic efficiency. Rex is a butterhead cultivar with thicker leaves than both Grand Rapids and Red Sails and tended to have the highest WUE. This cultivar-dependent difference in WUE could be important in nutrient management, but there was no difference in nutrient accumulation among cultivars despite the nutrient solution being designed for a WUE of 3 g L^-1^. This indicates that while there may be variations in WUE over time and cultivars, exact estimation of WUE is not necessary for the successful application of this nutrient management approach. When the CO_2_ is elevated in indoor environments, the WUE can be as high as 6 g L^-1^, and the solution concentration must therefore be increased to account for the increased ratio of growth to transpiration (Langenfeld et al., 2022).

### 4.3 No effect of rootzone calcium on tipburn

Tipburn is caused by Ca deficiency and leads to necrotic leaf margins, which make plants unsaleable. This issue is increased under high growth rates (typically from high light intensity) because rapid meristem growth exceeds the low meristem transpiration, which prevents adequate Ca from reaching the young tissue (Frantz et al., 2004).

The solution replacement and zero-discharge management approaches resulted in different Ca concentrations in the nutrient solution over time, but there was no difference in tipburn incidence between approaches. This supports the established hypothesis that Ca cannot be forced into lettuce plants by increasing the rootzone Ca concentration.

Tipburn was, however, cultivar dependent. Rex had significantly higher tipburn indices than Grand Rapids. Tipburn was minimal in Red Sails. In contrast to our results, Koyama et al. (2012) found leaf lettuce cultivars more susceptible than butterhead cultivars. This may be due to differences in specific cultivars among studies, which indicates that tipburn incidence cannot be generalized into broad lettuce growth habits.

### 4.4 Changes in EC and solution concentrations

The increasing EC in study one was mainly caused by the increase in solution concentration of macronutrients with passive uptake (Ca, Mg, and S). While B and Cu concentrations also increased in solution over time, their micromolar concentrations had a negligible effect on EC. The reduced EC changes in studies two and three were confirmed by monitoring the reduced accumulation of Ca, Mg, and S in the nutrient solutions. The EC measured in the latter crop cycles was lower than what is often maintained in commercial systems. Growth, however, is independent of EC because it is driven by individual nutrient concentrations. There were no nutrient deficiencies in plant tissues, and there was no increase in growth, which leads us to conclude that maintaining a higher EC does not benefit growth.

The EC of the Rex solution in study one remained below that of Grand Rapids and Red Sails due to the lower volume of solution added throughout the study. Holmes et al. (2019) saw a similar lower growth rate and higher heat tolerance of Rex compared to seventeen other lettuce cultivars grown in deep-flow hydroponics. This observation is consistent with the lower transpiration rate and lower solution volume requirement of Rex in our studies, which correlates with the increased tipburn discussed earlier. The lower concentrations of Ca and S in the Rex solution compared to Grand Rapids and Red Sails solutions throughout the study match the lower overall EC.

The three cultivars we studied had similar concentrations of nutrients remaining in solution following each harvest despite their different morphologies. This indicates that, within lettuce, cultivar differences in nutrition are minor.

As expected, nutrients with active uptake (N, P, and K) decreased rapidly in the nutrient solutions, which corresponded to a rapidly decreasing EC. Low nutrient concentrations can reduce antagonisms among elements. For example, high K concentrations can inhibit Ca uptake.

The increase in nutrient solution concentration of Ca, Mg, S, B, and Cu in study one indicated the concentration of these nutrients in solution could be reduced. We hypothesized that the healthy leaf tissue Ca concentration was 2%, but there was only 0.3% in the young leaf tissues across study one. When mature leaf tissue was analyzed from study three, the highest Ca concentrations were about 1.5%. It was not possible to “force feed” Ca into lettuce with higher concentrations in solution.

### 4.5 Adding ammonium through pH control

Controlling pH is essential in poorly buffered hydroponic solutions. Adding ammonium to the pH control solution provides low steady-state levels of ammonium in solution (Langenfeld and Bugbee, 2025). Nitrate is the dominant N form, but using only nitrate leads to a pH increase over time due to a net removal of protons from solution. Nitric acid and ammonium sulfate were combined in the pH control solution to reduce the rate of pH increase, but this resulted in the accumulation of sulfur in solution. We subsequently switched to a pH control solution of nitric acid and ammonium nitrate to minimize sulfur accumulation.

A voluminous body of literature has shown that partial substitution of nitrate with ammonium increases yield compared to nitrate alone, but the optimum ratio may be species dependent (Britto and Kronzucker, 2002). Weil et al. (2021) and Song et al. (2021) found that leaf-type lettuce yield increased when up to 50% of the N was supplied as ammonium. Savvas et al. (2006) found the head-type lettuce grew significantly larger when grown with 30% ammonium compared to no ammonium and de Souza Lauton Wenceslau et al. (2021) similarly found that yield was highest when ammonium percentages were below 23%.

Ammonium is also more rapidly absorbed from solution than nitrate, which leads to a net release of protons into solution and decreases pH. Previous studies in our laboratory indicated that this reduced the volume of pH control solution needed for a stable rootzone pH.

### 4.6 Nutrient concentrations in plant tissue

A common response to declining nutrient concentrations in solution is to reset them back to their initial concentrations. However, this approach can lead to excessive uptake of nutrients with active uptake. Bottoms et al. (2012) saw increased nitrate uptake in lettuce when supplied with excess N, but there was no change in growth. However, nitrate is a storage form of N in leaf tissue and can result in nitrate toxicity in humans (methemoglobinemia) at elevated levels (Santamaria, 2006). High concentrations of phosphorus in solution can lead to luxury uptake of phosphorus (Murphy et al., 1981). Broadley et al. (2002) identified about 75% of the P leaf tissue content in lettuce as being derived from luxury uptake as opposed to structural integration. Elevated phosphorus can cause Fe precipitation and deficiency (Parry and Bugbee, 2017) and Zn deficiency (Fageria, 2001). While the concentrations of N, P, K, and Mn rapidly declined during the first few plantings of each study, we measured steady levels within the plant tissues across plantings, which indicated that the initial set-point concentrations did not need to be maintained.

There was no trend of increasing or decreasing nutrient concentrations in the plant tissues among repeated plantings in any study, but there was random variability. For some nutrients this variability was the same among cultivars, but for other nutrients there was no pattern. The variability tended to be consistent among cultivar for nutrients with active uptake and inconsistent for nutrients with passive uptake.

### 4.7 Distribution of nutrients among plant tissues

The mass-balance approach is based on whole-plant tissue concentrations, so plants must be sampled appropriately to obtain total nutrient uptake. The standard sampling technique is to analyze nutrient contents in the uppermost fully expanded leaves, but young leaves do not represent the overall nutrient status of the plant. Analysis of both young and mature leaf tissues is necessary to determine total plant nutrient status.

Mobile nutrients [N, P, K, Mg, and S (Marschner et al., 1997)] are transported via the phloem and appear at their highest levels in the youngest leaves. Immobile nutrients [Ca (Hanger, 1979), B (Brown and Shelp, 1997), and Mn (Xu et al., 2006)] are not phloem mobile, typically accumulate in old leaves, and are at low levels in young leaves. These differences in mobility are consistent with our measurements for the distribution of these nutrients in plant tissue (**Table 5**). Calcium and manganese had the largest differences between young and mature leaf tissues and they are the two least mobile nutrients in lettuce.

Target tissue concentrations must be adapted if only young leaf tissue is sampled. The average young leaf tissue concentration of Ca was less than our target tissue concentration from our mass-balance calculation, and the concentration of Ca in solution increased over time, which indicates that plants were not Ca limited. If young leaf tissue were used as an indicator for Ca in lettuce, the target tissue concentration should be adjusted down. This would reduce the Ca concentration in the refill solution, which would reduce the accumulation of Ca in solution. A similar approach could be used for Mg. Since the average young leaf tissue concentration was about one half of the target concentration, and the concentration of Mg increased in solution over time, the Mg concentration should be reduced from 0.8 mM to 0.4 mM.

### 4.8 Mass-balance recovery

The mass-balance recovery of most nutrients in study three was between 90% and 100%, except for Zn, Mn, and Mo. The low concentrations of Mo found in the nutrient solutions makes analysis difficult, which may have led to its low mass balance recovery. The mass balance recovery for other elements, such as Ca, Fe, P, and Si, may have been reduced from interactions leading to precipitation. While Ca and P concentrations were kept low, and chelate was used to protect Fe, small amounts of precipitation could have removed elements from solution.

Zinc was the only nutrient with a mass-balance recovery consistently and significantly above 100%, which indicates zinc contamination. We used reverse osmosis water to prepare all the nutrient solutions, and the source water consistently measured below 0.001 ppm Zn. The expected Zn solution concentration was 0.20 ppm, but we consistently measured 0.24 ppm in fresh solution, which is 20% above our expected concentration and is the cause of the 120% mass-balance recovery of Zn. When this information is considered, the mass-balance recovery was 100% (a higher-than-expected input led to a higher-than-expected output). This contamination could be from a non-reagent grade chemical (chelated iron or potassium silicate). Our chelated iron contains about 0.005% Zn, but this would only be enough to increase the Zn concentration by 0.0002 ppm. The potassium silicate contains less than 1 ppm Zn by mass, which would not lead to a 20% error in our nutrient solution. Fortunately, this trace contamination of Zn was insufficient to cause Zn toxicity in any study.

The mass-balance recovery of Mn was often less than 40%, but Mn recovery in fresh solutions was 94%. This low recovery was caused by lower than expected nutrient solution concentrations as plant tissue concentrations were within expected ranges. The Mn concentrations in the nutrient solution at harvest were sometimes below our analytical detectable limit of 0.001 ppm, while the Mn concentrations in plant tissues were at sufficient levels. Savvas et al. (2009) measured rapid decreases in Mn in hydroponic tomato solutions. Tzerakis et al. (2013) reported a decrease in Mn solution concentration from 0.55 µM to 0.07 ppm after 21 d in hydroponic cucumber. Pace and Williams (2024) reported a Mn decrease from 0.70 to 0.29 ppm over 21 d in hydroponic lettuce. Rapidly disappearing elements from solution is evidence for active uptake, but if all Mn was recovered in the plant tissue, the concentration would have to be 130 ppm to achieve a mass-balance recovery of 100%.

Manganese may be precipitating from solution. Sonneveld and Voogt (1980) grew hydroponic tomatoes and found than Mn solubility was significantly higher at pH 5.0 – 5.5 than at pH 6.5 – 7.0. They also reported no growth differences between treatments, which indicates plants can obtain Mn even when some is precipitated. Bromfield (1978) found Mn-oxidizing bacteria in aerobic nutrient solutions at pH 6.5 and 8, but not pH 5. Our pH was maintained below 5.8, which should have minimized Mn oxidation. There could have been high pH micro-sites, especially as the plants matured, which may have led to Mn oxidation and the low mass-balance recovery, but the ample aeration should have minimized the potential for high pH microsites. It remains unclear why the recovery of Mn was low compared to all other essential elements in the nutrient solution.

### 4.9 Implications for hydroponic systems with different ratios of solution volume to cultivation area

The principle of mass balance holds regardless of the ratio of solution volume to cultivation area. A smaller volume of solution would reach steady-state equilibrium faster than a large volume but the final nutrient concentration in solution should not change.

### 4.10 Implications for soilless media systems

The principles and practices in this liquid hydroponic culture system are applicable to soilless media. These systems have a high rootzone surface area that enhances microbial activity and enables a higher fraction of ammonium in the nutrient solution. The cation exchange capacity of substrates additionally buffers nutrient availability over weeks and months.

The mass balance approach is based on the plant needs and the WUE, which are independent of substrate type. We have applied this approach to optimize yields in peat- and coir-based systems for many years*. Meeting plant needs without excess fertilization is the key to optimal nutrient management*.

### 4.11 Expanding to crops with a higher fraction of stems and fruits

Lettuce is a simple crop because it does not have a high stem fraction and does not have a reproductive phase. Optimal concentrations for elements in leaves are similar among species, and these results can thus be extrapolated to predict nutrient requirements during the early leaf growth of all species.

The ion concentration in solution likely should be decreased as plants mature because stems and fruits typically have lower nutrient requirements than leaves. Bugbee (2004) described these reduced nutrient requirements during reproductive growth for a wheat crop. This has been called dynamic nutrient management. As plants senesce, the WUE often decreases, which necessitates a further reduction of the ion concentration in the refill solution.

### 4.12 Differences with the original Hoagland and Arnon solutions

What is often referred to as “Hoagland solution” is a solution developed by Hoagland and Snyder (1933), modified by Hoagland and Arnon (1938) and revised by Arnon in 1950 (**Table 2**). The 1950 paper has over 21,000 citations listed by Google Scholar.

The nutrient concentrations in the original solutions are about double the solution concentrations used in this research (**Table 2**). The original solution was designed to use deionized water as the refill solution. As it became apparent that the refill solution should replace nutrients, the ion concentration in the original solution was often diluted by half to reduce ion accumulation in solution. This is commonly called “half-strength Hoagland solution”. This half strength solution has similar nutrient concentrations to the solution used in this study. Some of the nutrient elements (Ca, Mg and S) are still higher than necessary for nutrient uptake and the use of this solution would result in accumulation of these elements in solution.

Two notable differences are the concentrations of Zn and Cu. These elements were recommended at low levels in the original Hoagland solution because there was contamination of Zn and Cu from brass fittings in water supply lines. This contamination supplied an ample concentration of these two elements. The use of PVC and inert plastic valves has minimized ion contamination of water supply lines over the years, and a higher concentration of these two elements is now necessary in hydroponic solutions.

For a discussion of the original principles of nutrient solutions see Hoagland (1920).

### 4.13 Low nitrogen concentrations minimize volatile losses

The consistently high recovery of N in each study indicated minimal loss of volatile N from the rootzone. We recovered more than 90% of the N among plantings, which is higher than the 50% recovery found in field agriculture (Govindasamy et al., 2023). This high recovery is likely associated with the low concentration of N in solution. It has been previously demonstrated that high N concentrations in the rootzone are correlated with large volatile N losses. Reducing these losses represents a significant economic and environmental value of this mass-balance approach.

### 4.14 Limitations and future directions

The goal of this study was to examine nutrient solutions under a zero-discharge management approach. Lettuce was selected as a model crop due to its fast growth rate and ease of nutrient partitioning, and we expect our findings to be translatable to other species with primarily vegetative growth. Those with reproductive growth (fruiting crops) will likely require modifications across life cycle stages to match nutrient solutions to plant needs. Vegetative growth often decreases during reproductive growth, so fewer nutrients are often required as plants age. This concept was previously discussed by Bugbee (2004) when he proposed a three-stage nutrient solution for fruiting crops. Future work should therefore focus on optimizing nutrient recipes across life cycle stages for fruiting crops and replicating the results presented in this study to eliminate pseudoreplication.

This study was conducted using liquid hydroponics, which could limit its translatability to substrate-based hydroponic systems. Adsorption and desorption from solid substrates across crop cycles may complicate mass-balance recovery calculations. However, the principles of mass-balance remain valid in any closed system, which should enable the approach to be used across system types.

## 5 Conclusion

These data indicate that a mass-balance approach can be used to optimize a nutrient solution for lettuce growth. We found no difference in tissue nutrient concentrations or yield among lettuce cultivars when using this optimized solution in a zero-discharge management system compared to a solution replacement approach. Nutrients with active uptake quickly decreased in solution and did not need to be replenished, which shows that nutrient solutions can be recirculated across many cropping cycles without ion monitoring. This approach reduces fertilizer and water use, and increases nitrogen recovery, which improves economic viability and environmental sustainability.

## Data Availability

The original contributions presented in the study are included in the article/**Supplementary Material**. Further inquiries can be directed to the corresponding author.
