# Supplementary material for: Using mass-balance principles to optimize nutrition of lettuce
Source: Front Plant Sci. 2026 Aug 10;17:1885837. doi: 10.3389/fpls.2026.1885837 (PMC13500615; doi:10.3389/fpls.2026.1885837)
Supplement: Supplementary file 1 [file DataSheet1.pdf]

Photon conversion efficacy

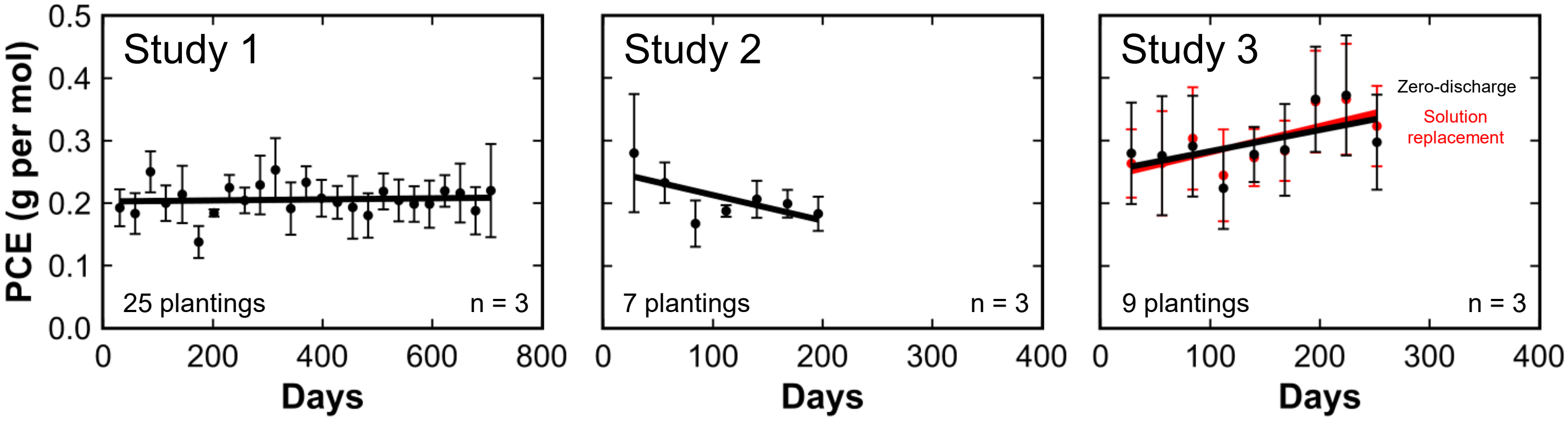

Supplemental Figure 1: The photon conversion efficacy across repeated plantings in three separate studies. The red line in study three indicates containers where the nutrient solution was completely replaced following each harvest. Error bars represent standard deviation, n = 3 lettuce cultivars.
