# Supplementary material for: Using mass-balance principles to optimize nutrition of lettuce
Source: Front Plant Sci. 2026 Aug 10;17:1885837. doi: 10.3389/fpls.2026.1885837 (PMC13500615; doi:10.3389/fpls.2026.1885837)
Supplement: Supplementary file 2 [file DataSheet2.pdf]

Study 1 – Nutrient solution (mg per L)

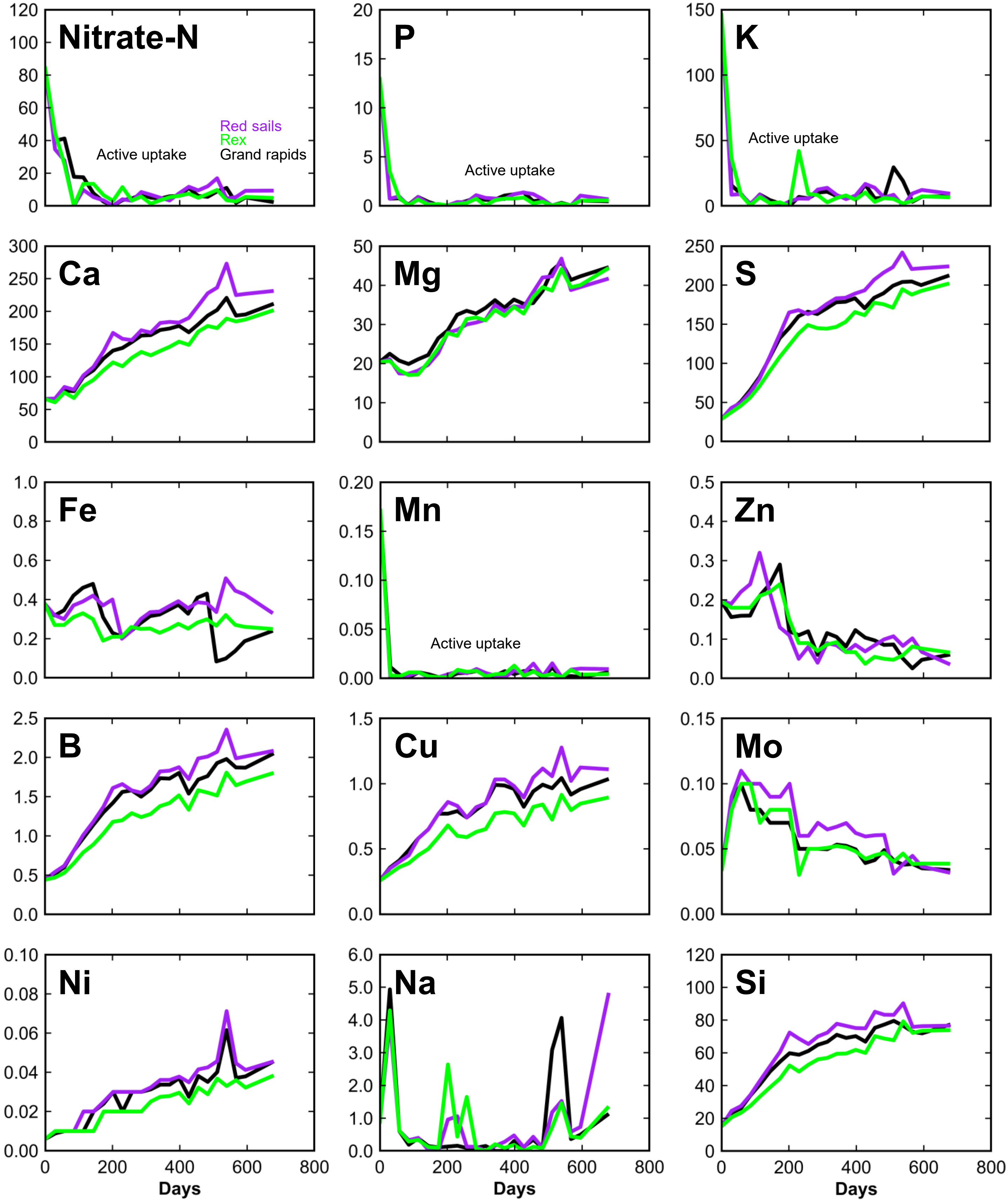

Supplemental Figure 2: The change in the solution concentration of nutrients in study one over the course of twenty-five repeated plantings in three lettuce (*Lactuca sativa*) cultivars: Grand Rapids (black), Red Sails (purple), and Rex (green).
