# Supplementary material for: Using mass-balance principles to optimize nutrition of lettuce
Source: Front Plant Sci. 2026 Aug 10;17:1885837. doi: 10.3389/fpls.2026.1885837 (PMC13500615; doi:10.3389/fpls.2026.1885837)
Supplement: Supplementary file 6 [file DataSheet6.pdf]

Study 1 – Root tissue

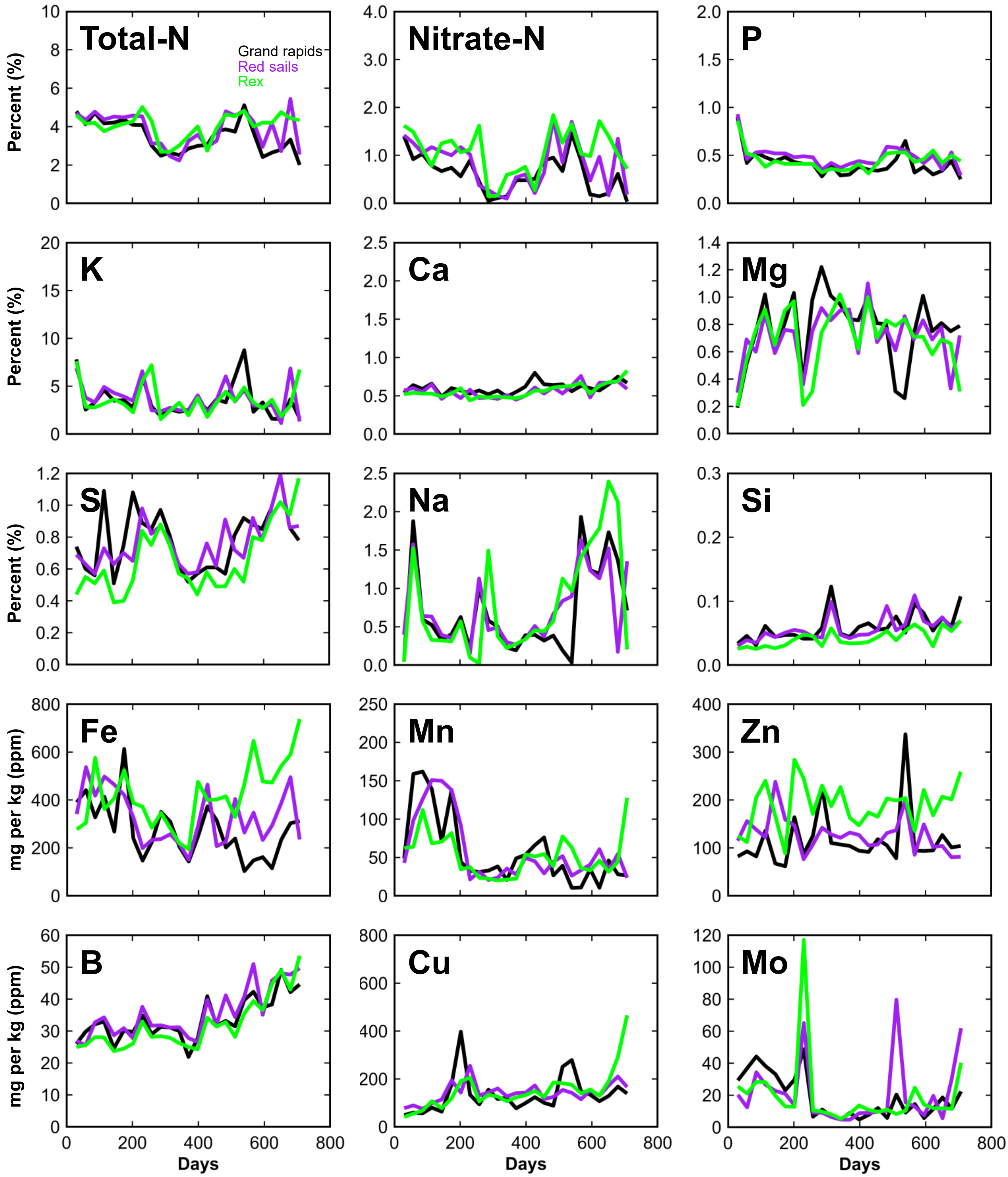

Supplemental Figure 6: The change in the root tissue concentration of essential nutrients in study one over the course of twenty-five repeated plantings in three lettuce (*Lactuca sativa*) cultivars: Grand Rapids (black), Red Sails (purple), and Rex (green).
